# Alteration of *p16* and *p15* genes in human uterine tumours

**DOI:** 10.1038/sj.bjc.6690379

**Published:** 1999-05-01

**Authors:** R Nakashima, M Fujita, T Enomoto, T Haba, K Yoshino, H Wada, H Kurachi, M Sasaki, K Wakasa, M Inoue, G Buzard, Y Murata

**Affiliations:** Department of Obstetrics and Gynecology, Osaka University Faculty of Medicine, 2-2, Yamadaoka, Suita, Osaka 565–0871, Japan; Department of Pathology, Osaka City University Hospital, 1–5–7, Asahi-machi, Abeno-ku, Osaka 545-0051, Japan; Department of Obstetrics and Gynecology, Kanazawa University Faculty of Medicine, 13–1, Takaramachi, Kanazawa, Ishikawa 920-0934, Japan; Intramural Research Support Program, SAIC Frederick, Bldg 538 Rm 205-D, NCI-Frederick Cancer Research and Development Center, Frederick, MD, 21702-1201, USA

**Keywords:** *p16*, *p15*, cervical carcinoma, endometrial carcinoma, methylation, immunohistochemistry

## Abstract

The roles of the *p16* and *p15* inhibitor of cyclin-dependent kinase tumour suppressor genes were examined in human uterine cervical and endometrial cancers. *p16* mRNA, examined by reverse transcription polymerase chain reaction (RT-PCR), was significantly reduced in five of 19 (26%) cervical and four of 25 (16%) endometrial tumours. Reduced expression of p16 protein, detected by immunohistochemistry, occurred even more frequently, in nine of 33 (27%) cervical and seven of 37 (19%) endometrial tumours. Hypermethylation of a site within the 5′-CpG island of the *p16* gene was detected in only one of 32 (3%) cervical tumours and none of 26 endometrial tumours. Homozygous *p16* gene deletion, evaluated by differential PCR analysis, was found in four of 40 (10%) cervical tumours and one of 38 (3%) endometrial tumours. Homozygous deletion of *p15* was found in three of 40 (8%) cervical tumours and one of 38 (3%) endometrial tumours. PCR-SSCP (single-strand conformation polymorphism) analysis detected point mutations in the *p16* gene in six (8%) of 78 uterine tumours (four of 40 (10%) cervical tumours and two of 38 (5%) endometrial tumours). Three were mis-sense mutations, one in codon 74 (CTG→ATG) and one in codon 129 (ACC→ATC), both in cervical carcinomas, and the other was in codon 127 (GGG→GAG) in an endometrial carcinoma. There was one non-sense mutation, in codon 50 (CGA→TGA), in an endometrial carcinoma. The remaining two were silent somatic cell mutations, both in cervical carcinomas, resulting in no amino acid change. These observations suggest that inactivation of the *p16* gene, either by homologous deletion, mutation or loss of expression, occurs in a subset of uterine tumours. © 1999 Cancer Research Campaign


*British Journal of Cancer* (1999) **80**(3/4), 458–467
© 1999 Cancer Research Campaign
Article no. bjoc.1998.0379

### Alteration of *p16* and *p15* genes in human uterine tumours

**R Nakashima[1], M Fujita[1], T Enomoto[1], T Haba[2], K Yoshino[1], H Wada[1], H Kurachi[1], M Sasaki[2], K Wakasa[2], M Inoue[3] G Buzard[4] and Y Murata[1]**

[1]Department of Obstetrics and Gynecology, Osaka University Faculty of Medicine, 2-2, Yamadaoka, Suita, Osaka 565–0871, Japan; [2]Department of Pathology, Osaka City University Hospital, 1–5–7, Asahi-machi, Abeno-ku, Osaka 545-0051, Japan; [3]Department of Obstetrics and Gynecology, Kanazawa University Faculty of Medicine, 13–1, Takaramachi, Kanazawa, Ishikawa 920-0934, Japan; [4]Intramural Research Support Program, SAIC Frederick, Bldg 538 Rm 205-D, NCI-Frederick Cancer Research and Development Center, Frederick, MD 21702-1201, USA

**Summary** The roles of the *p16* and *p15* inhibitor of cyclin-dependent kinase tumour suppressor genes were examined in human uterine cervical and endometrial cancers. *p16* mRNA, examined by reverse transcription polymerase chain reaction (RT-PCR), was significantly reduced in five of 19 (26%) cervical and four of 25 (16%) endometrial tumours. Reduced expression of p16 protein, detected by immunohistochemistry, occurred even more frequently, in nine of 33 (27%) cervical and seven of 37 (19%) endometrial tumours. Hypermethylation of a site within the 5′-CpG island of the *p16* gene was detected in only one of 32 (3%) cervical tumours and none of 26 endometrial tumours. Homozygous *p16* gene deletion, evaluated by differential PCR analysis, was found in four of 40 (10%) cervical tumours and one of 38 (3%) endometrial tumours. Homozygous deletion of *p15* was found in three of 40 (8%) cervical tumours and one of 38 (3%) endometrial tumours. PCR-SSCP (single-strand conformation polymorphism) analysis detected point mutations in the *p16* gene in six (8%) of 78 uterine tumours (four of 40 (10%) cervical tumours and two of 38 (5%) endometrial tumours). Three were mis-sense mutations, one in codon 74 (CTG→ATG) and one in codon 129 (ACC→ATC), both in cervical carcinomas, and the other was in codon 127 (GGG→GAG) in an endometrial carcinoma. There was one non-sense mutation, in codon 50 (CGA→TGA), in an endometrial carcinoma. The remaining two were silent somatic cell mutations, both in cervical carcinomas, resulting in no amino acid change. These observations suggest that inactivation of the *p16* gene, either by homologous deletion, mutation or loss of expression, occurs in a subset of uterine tumours.

**Keywords:** *p16*; *p15*; cervical carcinoma; endometrial carcinoma; methylation; immunohistochemistry

A large variety of human tumours manifest a heterozygous or homozygous deletion in the 9p21 chromosome region. The list includes malignant melanoma, glioma, lung, bladder, pancreatic and renal cancers (Kamb et al, 1994; Nobori et al, 1994), as well as gynaecological tumours (reviewed by Wong et al, 1997). Two putative tumour suppressor genes have been identified in this region: *p16* (also known as $p16^{INK4A}$, cyclin-dependent kinase 4 inhibitor, *CDK4I*, *CDKN2*, and *MTS1*), and *p15* ($p15^{INK4B}$).

The *p16* gene makes two different proteins, p16 and $p19^{ARF}$ (p19 alternative reading frame), using different overlapping reading frames, starting with different first exons. The p16 protein uses exon 1α, and $p19^{ARF}$ uses exon 1β; these two exons are alternatively spliced to the same second and third exons. The p16 and p15 proteins belong to a family of negative regulators of the cell cycle. Specific binding of p16 protein to the CDK4 or CDK6 cyclin-dependent protein kinases inhibits the phosphorylation activity of CDK–cyclin D complexes towards the nuclear RB/E2F protein complex (Serrano et al, 1993). p16 normally prevents phosphorylation of RB, resulting in RB's retention of E2F. Failure to release E2F at the late G1 checkpoint blocks the cell from entering the S phase (Hengstschläger et al, 1996; Lukas et al, 1996). The $p19^{ARF}$ protein, although it has an unrelated amino acid sequence, has cell cycle arresting functions. It is the p16 protein that now appears to be the major target of mutations and deletions at the 9p21 loci.

The *p15* (*MTS2*) putative tumour suppressor gene is located 25 kb centromeric of the *p16* gene on 9p. *p15* contains sequences highly homologous to exon 2 of *p16* and, like *p16*, inhibits both CDK4 and CDK6 kinase activities (Guan et al, 1994; Hannon and Beach, 1994). Homozygous deletions of *p15* and hypermethylation-associated loss of *p15* expression have been reported in glioblastomas (Jen et al, 1994).

The *p16*/$p19^{ARF}$ and *p15* genes appear to play variably important roles in human tumorigenesis, with critical tissue specificities and uncertain implications for clinical prognoses. Little is currently known about the potential role of these genes in reproductive tract biology, and specifically in uterine tumours. We have begun to examine the expression of the *p16* gene at the level of mRNA and protein, the methylation status of the 5′-CpG island of *p16* exon 1α, and for *p16* point mutations and homozygous deletions in these tumours. We have also analysed the *p15* gene for mutations and homozygous deletions. We report that the inactivation of the *p16* gene, either by homozygous gene deletion, mutation or loss of protein expression, occurs in a small but significant subset of these uterine tumours.

*Received 5 March 1998*
*Revised 2 October 1998*
*Accepted 21 October 1998*

*Correspondence to:* T Enomoto

### MATERIALS AND METHODS

### Tissue samples

Samples used in this study were randomly obtained from patients who had been admitted to the Department of Obstetrics and Gynecology at the Osaka University Hospital in Osaka, Japan. Informed consent was obtained from all patients. No initial chemotherapy or radiation therapy was performed prior to tumour sampling. Tissues were mostly obtained by hysterectomy, except for those cases with advanced clinical stages in which surgery was not performed as the primary treatment. Tissues were sampled for histopathological diagnosis, and remainders were quick frozen for later extraction of RNA and DNA. We evaluated a total of 19 cervical tumours (16 squamous cell carcinomas and three adenocarcinomas) and a total of 25 endometrial tumours (22 endometrioid adenocarcinomas and three complex hyperplasia with atypia) for *p16* mRNA expression. RNA was extracted with guanidinium isothiocyanate, followed by centrifugation in a caesium chloride solution. RNA was also extracted from histologically normal tissues (two ovaries, four cervixes and four endometrium). High molecular weight DNA was extracted from 40 cervical tumours (35 squamous cell carcinomas and five adenocarcinomas) and 38 endometrial tumours (30 endometrioid adenocarcinomas and eight complex hyperplasias with atypia) following the procedures previously described (Enomoto et al, 1991).

### RT-PCR analysis

For cDNA synthesis, 1 μg of total cellular RNA was annealed with random hexamers at 26°C for 10 min. The RNA was transcribed with 10 units of AMV reverse transcriptase (Gibco-BRL, Rockville, MD, USA) at 42°C for 90 min in the presence of the ribonuclease inhibitor RNasin (1 μl; Promega, Madison, WI, USA). A cDNA aliquot corresponding to 200–500 ng of RNA was used as the template for polymerase chain reaction (PCR) amplification. Primers used were 5′-TTATTTGAGCTTTGGTTCTG-3′ for the *p16* antisense primer, which corresponds to nucleotides 894–913 in the cDNA sequence, and 5′-CCCGCTTTCG-TAGTTTTCAT-3′ for the sense primer, which corresponds to 559–578 in the cDNA sequence. For control of reverse transcription (RT)-PCR, a 319-bp fragment of β-*actin* cDNA was also amplified using primers previously described (Fuqua et al, 1990).

The PCR reactions for all the following experiments were in a 25 μl volume. This particular PCR reaction mixture contained cDNA (200–500 ng), 0.5 μM of each primer for *p16*, or 0.05 μM of each primer for β-*actin*, and PCR buffer (100 μM of each deoxynucleotide triphosphate, 1.5 mM magnesium chloride, 50 mM potassium chloride, 10 mM Tris-HCl pH 8.3 and 0.01% gelatin). All the PCR reactions in this manuscript were initially denatured at 94°C for 5 min, then 0.5 U of *Taq* polymerase (Perkin-Elmer Cetus, Norwalk, CT, USA) was added. One amplification cycle for the *p16* fragment consisted of 1 min each at 94°C, 58°C and 72°C. One cycle for the β-*actin* fragment consisted of 1 min each at 94°C, 50°C and 72°C. A total of 28 cycles of PCR amplification was performed. PCR products were fractionated in 2.5% agarose gels, and visualized by staining with ethidium bromide. Band quantitation was done by densitometric analysis.

### Immunohistochemistry

The tissue localization of p16 protein was determined immunohistochemically by the labelled streptavidin–biotin method on formalin-fixed paraffin-embedded tissues. Sections were placed on glass slides coated with 0.02% poly-L-lysine. Deparaffinized sections were immersed in 10 mM sodium citrate buffer (pH 6.0) and autoclaved at 121°C for 15 min. The immuno-detection procedures were carried out according to the manufacturer's recommendations. A polyclonal rabbit anti-human p16INK4 protein antibody (1:100 dilution; PharMingen, San Diego, CA, USA) was used as the primary antibody. Sections from normal ovary and normal proliferative endometrium were used as positive controls. Sections incubated with normal rabbit pre-immune serum, instead of the corresponding primary rabbit antibody, were used as negative controls. The relative number of immunoreactive cells was scored from a negative reaction to a grade 2(+) staining for p16, as follows: (–, no staining was observed in any tumour cell; ±, less than 50% of the tumour cells were stained positively; +, more than 50% of the tumour cells were stained positively).

### Analysis of the methylation status of the *p16* gene

The methylation status of the *p16* gene promoter region 5′-CpG island of which extends 3′ into the first exon, was analysed using multiplex PCR analysis, as described by Lo et al (1996). One μg of high molecular weight DNA was digested with 10 U of the methylation-sensitive restriction enzyme *Sma* I at 30°C for 12 h, then another 10 U of *Sma* I was added and digestion was continued for an additional 12 h. The digested DNA was then subjected to multiplex PCR analysis. Primers used were the 2F and 1108R primers described by Kamb et al (1994), which amplify exon 1 of the *p16* gene, including a methylation-status diagnostic *Sma* I site. As an internal control, the sequence tagged site (STS) 1063.7 on the distal side of the *p16* gene was also co-amplified. The high molecular weight DNA was alternatively digested with 10 U of the methylation-sensitive enzyme *Eag* I at 37°C for 12 h, then another 10 U of *Eag* I was added and digestion was continued for an additional 12 h. PCR amplification was performed as described above. Undigested tumour DNA was amplified as an additional control.

### Detection of homozygous deletion of *p16* gene by differential PCR analysis

To detect homozygous deletion of the *p16* gene, a 111-bp fragment of exon 2–intron 2 was amplified by PCR. Primers used were 5′-CAAATTCTCAGATCATCAGTCCT-3′ for the *p16* antisense primer, which corresponds to nucleotide 502–524, and 5′-GATGTCGCACGGTACCTG-3′ for the sense primer, which corresponds to nucleotide 414–431. For internal control of PCR, we simultaneously amplified a 136-bp fragment in the promotor region of the transferrin receptor gene. Primers used were 5′-CAGGATGAAGGGAGGACAC-3′ for the transferrin receptor gene antisense primer, which corresponds to nucleotides 405–423, and 5′-GCTATAAACCGCCGGTTAG-3′ for the sense primer, which corresponds to nucleotides 288–306 (Owen and Kuëhn, 1987). The PCR reaction mixture contained genomic DNA (0.1 μg), 0.1 μM of each primer for *p16*, 0.1 μM of each primer for the transferrin receptor gene and PCR buffer. One cycle of PCR consisted of 30 s each at 94°C and 60°C. A total of 25 cycles of PCR was performed. The PCR products were separated by electrophoresis on a 12% polyacrylamide gel and the bands were visualized by ethidium bromide staining under UV light and photographed.

© Cancer Research Campaign 1999

**Table 1** Alterations of *p16* in uterine tumours

| Histology | Mutation/Deletion | Hypermethylation | Loss of expression | |
|---|---|---|---|---|
| | | | mRNA | Immunohistochemistry |
| Cervical tumours | 8/40 (20%) | 1/32 (3%) | 5/19 (26%) | 9/33 (27%) |
| Squamous cell carcinoma | 8/35 (23%) | 1/27 (4%) | 4/16 (25%) | 6/28 (21%) |
| Adenocarcinoma | 0/5 (0%) | 0/5 (0%) | 1/3 (33%) | 3/5 (60%) |
| Endometrial tumours | 3/38 (8%) | 0/26 (0%) | 4/25 (16%) | 7/37 (19%) |
| Complex hyperplasia with atypia | 0/8 (0%) | 0/6 (0%) | 0/3 (0%) | 1/7 (14%) |
| Endometrioid adenocarcinoma | 3/30 (10%) | 0/20 (0%) | 4/22 (18%) | 6/30 (20%) |

### Detection of *p16* and *p15* gene mutation by PCR-SSCP analysis

Exons 1–3 of the *p16* gene and exon 2 of the *p15* gene were independently amplified by two-step PCR. Primers used for the amplification of the *p16* gene fragment were as follows: 5′-CGGAGAGGGGGAGAGCAG-3′ and 5′-TCCCCTTTTTC-CGGAGAATTCG-3′ for exon 1 (Marchetti et al, 1997); 5′-TCTGAGCTTTGGAAGTTCG-3′ and 5′GGAAATTGAAA-CTGGAAGC-3′ for exon 2 (Kamb et al, 1994), and 5′-AGGAATTCGGTAGGGACGGCAAGAGAGG-3′ and 5′-GAAG-CTTGGGGGAAGGCATATATCTACG-3′ for exon 3 (Marchetti et al, 1997). Primers used for the amplification of the *p15* gene were 5′-TGGCTCTGACCACTCTGC-3′ for the sense primer, which corresponds to nucleotides 61–78, and 5′-AGCGAATTCGGGTGGGAAATTGGGTAAGAA-3′ for the antisense primer, which corresponds to nucleotides 397–426. Sequences for the primers were described previously by Washimi et al (1995). The PCR reaction mixture contained 0.1 μg of genomic DNA, 0.1 μM of each primer, PCR buffer and 5% dimethyl sulphoxide (DMSO). One cycle of PCR for *p16* consisted of 45 s at 94°C (denaturing), 45 s at 50°C for exons 1 and 3 and 45°C for exon 2 (annealing), and 30 s at 72°C (elongation). One cycle of PCR for *p15* consisted of 45 s at 94°C, 30 s at 52°C and 30 s at 72°C. A total of 30 cycles of amplification were performed. Aliquots (10 μl) of the PCR product were assessed by 2% agarose gel electrophoresis to confirm amplification of the target. The second step of PCR was performed using 1 μl of the first step product with 0.05 μl [α-$^{32}$P]-dCTP (370 MBq ml$^{-1}$), 0.05 μM of each primer for each gene, independently, 25 mM of each deoxynucleotide triphosphate, and 0.1 U of *Taq* polymerase in a 4 μl total volume, for 10 cycles.

PCR product (2 μl) of exon 2 of *p16* or *p15* was digested with 10 U of *Sma* I (Toyobo, Japan) at 30°C in 10 μl of the digestion buffer recommended by the manufacturer. The PCR product of *p16* was alternatively digested with *Hae* II and *EcoN* I (10 U each) at 37°C. Digestion of the 509-bp fragment of *p16* with *Sma* I yielded two fragments (260-bp and 249-bp), digestion with *Hae* II and *EcoN* I yielded three fragments (171-bp, 182-bp and 156-bp). Digestion of the 366-bp fragment of *p15* with *Sma* I yielded two fragments (149-bp and 217-bp). After incubation for 12 h, digestion was terminated by incubating the mixture at 95°C for 10 min. Then two volumes of a gel loading buffer (95% formamide, 20 mM EDTA, 0.05% bromphenol blue and 0.05% xylene cyanol) were added. The DNA was heat-denatured at 98°C for 10 min. Two microliters of this mixture was immediately loaded on an 8% non-denaturing polyacrylamide gel and electrophoresed at a constant 600 V at 25°C. The gel was vacuum dried and exposed to Kodak X-Omat film at room temperature for 24 h.

### Analysis of *p16* gene mutations by sequencing

We purified the PCR-amplified *p16* gene fragments on a 2% agarose gel and subcloned them into the *Sma* I site of pUC19. A mixture containing at least 10 subclones selected by colony PCR was used as the template for DNA sequencing. Sequencing was performed by the dideoxy termination method using a Sequenase 2.0 kit (USB, Cleveland, OH, USA).

### Statistical analysis

The significance of differences in the frequency with which gene alterations occurred in different categories of lesions was estimated using the $\chi^2$ test.

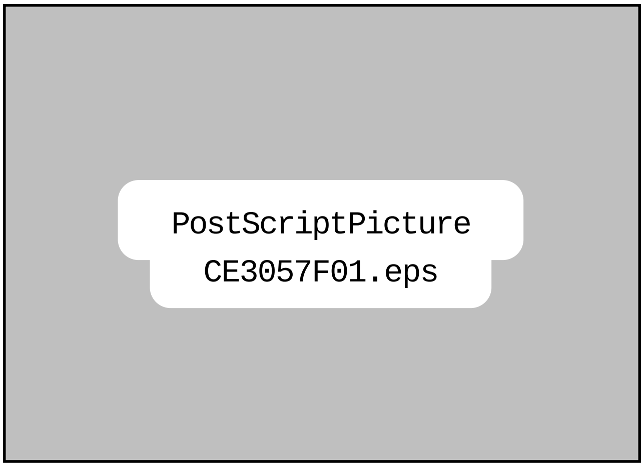

**Figure 1** RT-PCR analysis demonstrates the loss of expression of *p16* mRNA. RT-PCR generated a 355-bp fragment of the *p16* gene **(A)** and a 319-bp fragment of the β-actin gene **(B)**. PCR products were fractionated in 2.5% agarose gels and visualized by staining with ethidium bromide. Note that the 319-bp fragment of β-actin was successfully generated in all the samples analysed **(B)**, whereas fragments of *p16* were not amplified in lanes 2, 6, 12, 14 and 15, suggesting the loss of *p16* mRNA expression in these tumours. Lane 1, normal endometrium; lanes 2–7, endometrial carcinoma; lane 8, normal cervix; lanes 9–16, cervical carcinoma

 *© Cancer Research Campaign 1999*

### RESULTS

### Loss of *p16* mRNA expression

Expression of *p16* mRNA was examined in a total of 44 uterine tumours by RT-PCR analysis, and compared to that of histologically normal cervical epithelium and endometrium from four uteri. A 355-bp fragment of the *p16* gene was generated (Figure 1A).The 319-bp fragment of β-actin was amplified independently as a control (Figure 1B). We successfully amplified both fragments in all the histologically normal tissues, suggesting that *p16* mRNA is expressed to some extent in the normal uterine cervix and endometrium. The 319-bp fragment of the β-*actin* gene was successfully generated in all 19 cervical tumours and 25 endometrial tumours. On the other hand, the only slightly larger 355-bp fragment of the *p16* gene was amplified minimally, or not at all, in five of 19 (26%) cervical tumours and four of 25 (16%) endometrial tumours. By cervical tumour sub-type, four of 16 (25%) squamous cell carcinomas and one of three (33%) adenocarcinomas had little or no *p16* mRNA. For endometrial tumours, four of 22 (18%) endometrioid adenocarcinomas lacked *p16* mRNA (Table 1). The three complex hyperplasia with atypia tumours each had *p16* mRNA. This cumulative data suggests that *p16* mRNA expression was greatly reduced or absent in a small but significant fraction of human uterine tumours.

### Immunohistochemical detection of the *p16* gene product

The p16 protein was examined immunohistochemically in 33 uterine cervical tumours and 37 endometrial tumours (Figure 2). While normal cervical epithelium and endometrium from four uteri showed positive p16 staining, both in the nucleus and cytoplasm, nine cervical tumours (27%) and seven endometrial tumours (19%) showed no staining or only weak staining, with the exception of positively staining lymphoid cells (Table 1). This included six of 28 (21%) squamous cell carcinomas and three of five (60%) adenocarcinomas of the uterine cervix, and six of 30 (20%) endometrioid adenocarcinomas and one of seven (14%) complex hyperplasia with atypia of the uterine corpus. The intensity of the p16 immunohistochemical staining correlated significantly with the level of p16 mRNA expression ($P < 0.005$ by the $\chi^2$ test).

### Methylation status of the 5′-CpG island of *p16*

Methylation of the 5′-CpG island of the *p16* gene was analysed by multiplex PCR amplification. PCR amplification from undigested normal tissue DNA yielded a 340-bp fragment of exon 1 of the *p16* gene together with the 200-bp fragment of an internal control (1063.7) fragment. DNA samples were then digested with the methylation-sensitive restriction endonuclease *Sma* I and were then PCR amplified. The 340-bp fragment of the exon 1 of the *p16* gene could not be amplified if the *Sma* I site was unmethylated. In contrast, the presence of the 340-bp fragment indicated that the *Sma* I site was methylated (Figure 3). Of 58 uterine tumours analysed, only one of 32 (3%) cervical tumours, and none of the 26 endometrial tumours, showed apparent hypermethylation of this CpG island site. Digestion with the methylation-sensitive restriction enzyme *Eag* I also suggested the presence of hypermethylation in only one cervical tumour, one in which hypermethylation at the *Sma* I site was also previously demonstrated (data not shown).

### Homozygous deletion of the *p16* gene

Deletion of the *p16* gene was examined by differential PCR analysis of a total of 40 cervical tumours and 38 endometrial tumours. PCR amplification was performed to simultaneously generate a 111-bp fragment of the exon 2–intron 2 region of the *p16* gene and a 136-bp fragment of the promoter region of the transferrin receptor gene. The larger transferrin receptor gene fragment, which served as an internal PCR control, was successfully amplified in all 78 cases, whereas the intensity of the *p16* gene PCR product in five uterine tumours was remarkably reduced compared to the transferrin receptor gene PCR product (data not shown). Four were squamous cell carcinomas of the uterine cervix, and one was an endometrioid adenocarcinoma of the uterine corpus. We therefore presume that these five cases contained a homozygous deletion of the *p16* gene. The residual signal was presumably from wild-type stromal and infiltrative cells.

### Mutations in the *p16* gene

We screened a total of 78 uterine epithelial tumours for *p16* mutations by PCR-SSCP (single-strand conformation polymorphism) analysis. Bands with mobility shifts indicating the presence of mutation were not observed in either exon 1 or exon 3 of the *p16* gene in any of 78 uterine epithelial tumours. However, aberrant bands were observed in exon 2 of the *p16* gene in six of 78 uterine tumours; four of 40 (10%) cervical tumours and two of 38 (5%) endometrial tumours. Figure 4 shows an example of multiplex–SSCP analysis after *Sma* I digestion. In these six cases, we observed one or two bands with mobility shifts that were clearly distinguishable from the normal alleles, whereas we saw only the normal allele bands in the remaining 72 cases. Samples which showed aberrant bands after *Sma* I digestion also showed bands after *Hae* II and *EcoN* I digestion, whereas none of the remaining 72 tumours did.

Direct sequencing further defined the mutations in the *p16* gene. Sequencing of two or more independently PCR-amplified and purified DNAs was performed by the dideoxy method (Figure 5). Single-base substitutions were observed in all six cases. In squamous cell carcinomas of the uterine cervix, mutations identified were: a mis-sense CTG→ATG mutation (Val→Met) in codon 74; a mis-sense ACC→ATC mutation (Thr→Ile) in codon 129; and two silent mutations (GCG→GCA in codon 94 and GGT→GGC in codon 142) resulting in no amino acid change. In endometrioid adenocarcinomas of the uterine corpus, a non-sense CGA→TGA mutation (Arg→stop) in codon 50, and a mis-sense GGG→GAG mutation (Gly→Glu) in codon 127 were detected. Only the non-sense stop codon at codon 50 has been previously described in the database, occurring in a melanoma (Foulkes et al, 1997; Pollock et al, 1996).

### Mutations and homozygous deletions of the *p15* gene

We screened a total of 78 uterine epithelial tumours for mutations in exon 2 of the *p15* gene. We PCR amplified a 366-bp fragment which contained the entire exon 2 of the *p15* gene and obtained a product from most of the tumours. However, three squamous cell

© Cancer Research Campaign 1999

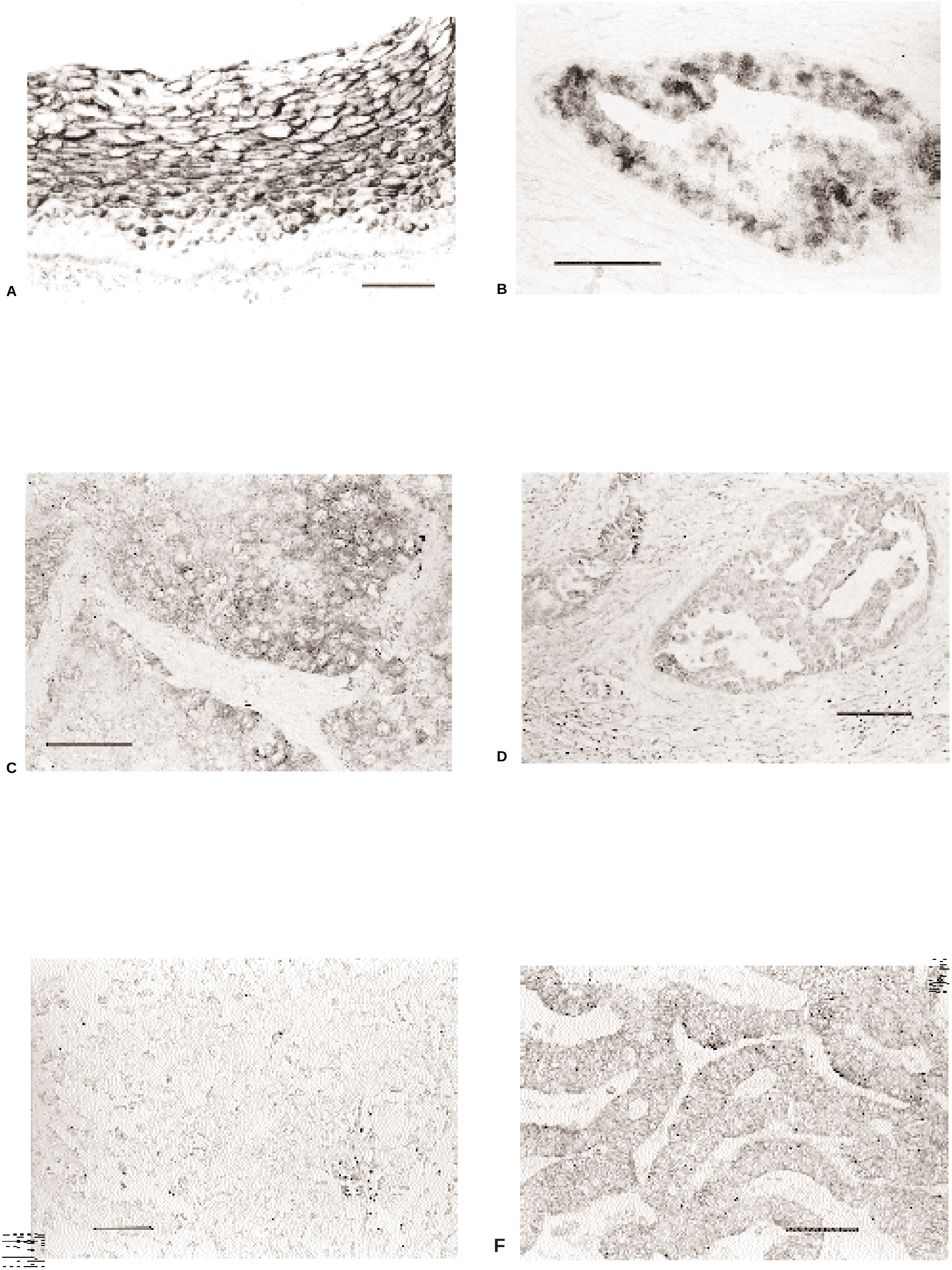

*© Cancer Research Campaign 1999*

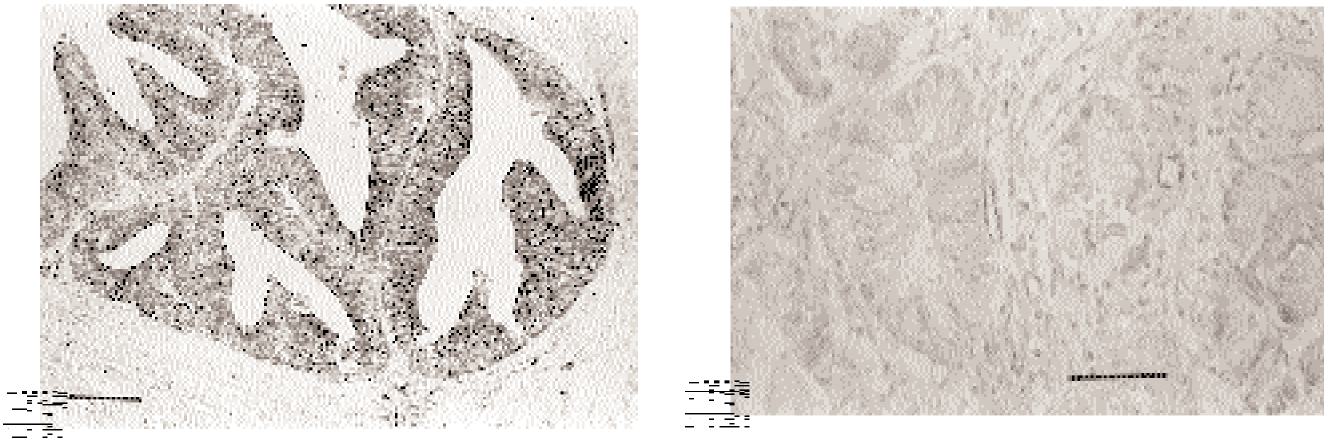

**Figure 2** An immunohistochemical analysis of the *p16* gene product finds that normal squamous epithelium of the uterine cervix **(A)** and normal endocervical glands **(B)** show positive staining, whereas the adjacent normal mesenchymal tissue shows negative staining. A squamous cell carcinoma of the uterine cervix shows positive staining **(C)**. Endocervical adenocarcinomas with positive staining **(D)** and negative staining **(E)**. Endometrioid adenocarcinomas of the uterine corpus with positive staining (**F** and **G**) and negative staining **(H)**. Scale bars, 0.1 mm in **(A)**, **(C)**, **(D)** and **(E)**, and 0.05 mm in **(B)**, **(F)**, **(G)** and **(H)**

carcinomas of the uterine cervix and one endometrioid adenocarcinomas of the uterine corpus, which we found to also have a homozygous deletion of the *p16* gene, repetitively failed to yield this *p15* PCR fragment. This suggested that in these tumours the homologous deletion of the *p15* gene extended through the neighbouring *p16* gene. We digested the PCR fragment of the *p15* gene with *Sma* I and subsequently analysed for the presence of mutations by SSCP analysis. Aberrant bands indicative of point mutations were not observed in any of the 40 cervical tumours or 38 endometrial tumours.

### DISCUSSION

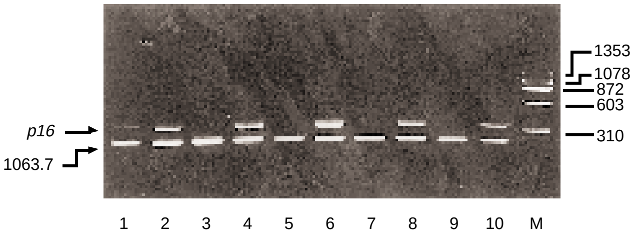

**Figure 3** Analysis of the methylation status of the *p16* gene by multiplex PCR. The 340-bp fragment of exon 1 of the *p16* gene and the STS marker 1063.7 were co-amplified. The *Sma* I-digested (lanes 1, 3, 5, 7 and 9) and undigested DNA (lanes 2, 4, 6, 8 and 10), derived from cervical carcinomas, endometrial carcinomas and normal endometrium, were used as templates. Lanes 1–2, squamous cell carcinoma of the uterine cervix; lanes 3–4, an endocervical adenocarcinoma; lanes 5–6, a grade-1 endometrioid adenocarcinoma of uterine corpus; lanes 7–8, a grade-3 endometrioid adenocarcinoma of the uterine corpus; lanes 9–10, normal endometrium. Pretreatment with *Sma* I blocked the amplification of the *p16* gene fragment from an endocervical adenocarcinoma (lane 3), and in two endometrioid adenocarcinomas (lanes 5 and 7) and a normal endometrium (lane 9), suggesting that the *Sma* I site is unmethylated in these tumours and in normal endometrium. In contrast, the *p16* gene fragment was amplified regardless of the pretreatment with *Sma* I in a squamous cell carcinoma of the uterine cervix (lane 1), suggesting that *p16* is at least partially methylated and therefore protected from digestion

In the present study, we have explored the involvement of the *p16* and *p15* genes in various types of human uterine tumours. None of 78 tumours had a *p15* point mutation. Four tumours shared deletion of the adjoining *p15* and *p16* genes. We show that *p16* inactivation by homologous deletion or point mutation occurs relatively more frequently in squamous cell carcinomas in the uterine cervix (23%) than in the adenocarcinomas of the uterine endocervix or corpus (0% and 10% respectively). We observed mutations only in exon 2 of the *p16* gene, which is in accordance with findings in other types of human primary tumours, where exon 1 had only 30 mutations compared to 105 reported in exon 2, as reviewed by Foulkes et al (1997).

Our findings should be considered in light of previous reports regarding *p16* and/or *p15* in human uterine tumours (Hatta et al, 1995; Kelly et al, 1995; Peiffer et al, 1995; Hirama et al, 1996; Wong et al, 1997; Kim et al, 1998; Munirajan et al, 1998). Hatta et al (1995) found no mutations, deletions, or rearrangements in 15 endometrial carcinomas. Kelly et al (1995) found no homozygous *p16* deletions or point mutations, and no loss of abundant p16 protein expression in 11 cervical cancer cell lines. Peiffer et al (1995) found 9p21 loss of heterozygosity (LOH) in three of 34 endometrioid tumours, and two tumours which had point mutations, only one of which was accompanied by LOH. Hirama et al (1996) found no deletion or mutation of *p16* in 41 primary cervical tumours or eight cell lines. Wong et al (1997) found homologous deletions of *p16* in seven of 128 (5%) cervical carcinomas (histological type not specified) and in one of 41 (2%) endometrial carcinomas, and they found homozygous deletions of *p15* in 19 of 128 (15%) of cervical carcinomas, and in one of 41 (2%) endometrial carcinomas. Wong et al, found no somatic *p16* point mutations in any of the 128 cervical or 41 endometrial carcinomas. Kim et al (1998) found no mutations or homozygous deletions of *p15* or *p16* in 57 cervical carcinomas. Munirajan et al (1998) found no *p16* mutations in 43 cervical carcinomas.

The incidence of mutations or deletions of *p16* in uterine tissues in various manuscripts may differ due to several factors. When one analyses primary tumours, contamination with normal stromal cells or inflammatory cells can cause an underestimation of gene deletions or mutations. The methods used to evaluate the mutations may also have different sensitivities. Sheffield et al (1993)

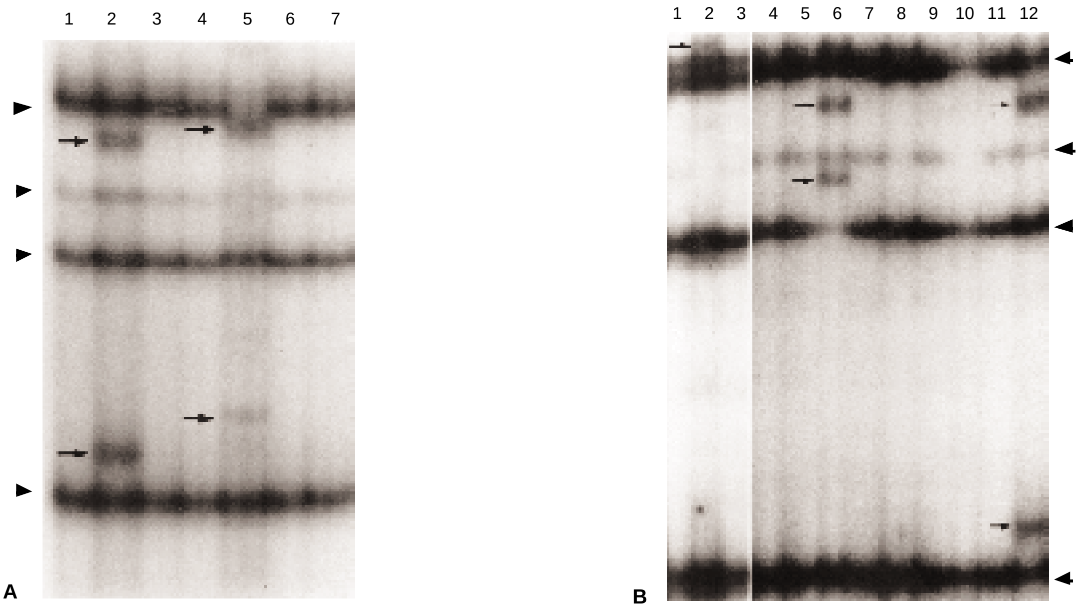

**Figure 4** Detection of *p16* gene mutations in cervical carcinoma **(A)** and in endometrial carcinoma **(B)** by multiplex–SSCP analysis is shown. The 509-bp fragment surrounding exon 2 of the *p16* gene was PCR amplified, with incorporation of [α-$^{32}$P]-dCTP. The PCR product was digested with *Sma* I, heat-denatured and electrophoresed in an 8% non-denaturing polyacrylamide gel at 25°C. The digested PCR products from wild-type *p16* sequences yielded four bands (arrowheads). In lanes 2 and 5 in **(A)** and in lanes 2, 6 and 12 in **(B)**, one or two bands with mobility shifts (arrows) are observed, suggesting the presence of a mutation have suggested that the most important parameter for determining the sensitivity of SSCP analysis was the size of the fragment. They found that the optimally sized fragment for sensitive base substitution detection was approximately 150 bp, at which 95% of mutations were detected. The sensitivity decreases with increases in the size of the fragment. We have cleaved the 509-bp fragment of exon 2 into two fragments by *Sma* I digestion, and into three fragments (171 bp, 182 bp and 156 bp) by *Hae* II and *Eco*N I digestion, before SSCP analysis in order to achieve the best sensitivity and to have overlapping looks for mutations. Most of the previous studies used larger and/or non-overlapping fragments for their SSCP analysis, with presumably less sensitivity. The reason for the higher incidence of mutations or deletions of *p16* in this manuscript compared to the previous reports may thus be attributed to the smaller size and/or more rigorous analysis of our fragments.

Most of these previous reports did not fully explore the expression levels of the *p16* mRNA and/or protein in uterine tumours. We show that two conventional mechanisms of *p16* gene inactivation which occur frequently in other tumour types, i.e. homozygous deletion or point mutation, occur only rarely in

**Table 2** Uterine tumours with loss of *p16* expression

| Histology | | IHC[a] | mRNA | Mutation/Deletion | Hypermethylation |
|---|---|---|---|---|---|
| Cervical tumours | | | | | |
| SCC | LCNK | – | – | DEL | NA |
| SCC | LCNK | – | – | DEL | NA |
| SCC | LCNK | – | – | DEL | NA |
| SCC | LCNK | – | NT | DEL | NA |
| SCC | K | – | + | WT | – |
| SCC | K | – | – | WT | + |
| AC | G1 | – | – | WT | – |
| AC | G1 | – | + | WT | – |
| AC | G3 | ± | + | WT | – |
| Endometrial tumours | | | | | |
| AC | G1 | – | – | DEL | NA |
| AC | G1 | – | – | NON-SENSE | – |
| AC | G2 | – | – | WT | – |
| AC | G3 | – | NT | WT | – |
| AC | G2 | – | – | WT | NT |
| AC | G1 | ± | + | WT | – |
| CH-A | | – | + | WT | – |

[a]IHC, immunohistochemistry; SCC, squamous cell carcinoma; AC, adenocarcinoma; CH-A, complex hyperplasia with atypia; LCNK, large-cell non-keratinizing type; K, keratinizing type; G, histological grade; NT, not tested; DEL, deletion; WT, wild-type; NA, not analysed because *p16* is deleted.

 © Cancer Research Campaign 1999

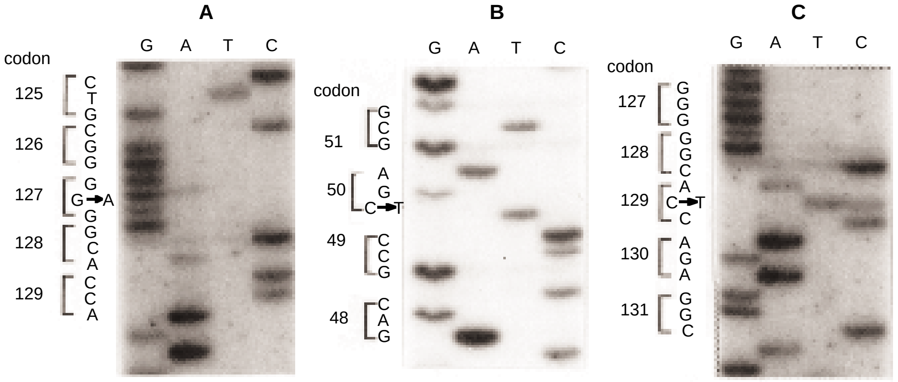

**Figure 5** Mutations in the *p16* gene are demonstrated by sequencing. **(A)** A GGG→GAG transition in codon 127 resulting in Gly→Glu in a case of endometrioid adenocarcinoma of the uterine corpus; **(B)** a CGA→TGA transition in codon 50 resulting in new stop codon in a case of endometrioid adenocarcinoma of the uterine corpus; **(C)** an ACC→ATC transition in codon 129 resulting in Thr→Ile in a case of squamous cell carcinoma of the uterine cervix adenocarcinomas of the uterine endocervix or endometrium. However, we do find that expression of the mRNA and/or protein from the intact *p16* gene is frequently decreased or undetectable in many of these tumours. We have observed an absence of *p16* mRNA expression in 33% of the endocervical adenocarcinomas and 18% of the endometrial adenocarcinomas.

We found that aberrant expression of the p16 protein is observed in 60% of endocervical adenocarcinomas and 20% of endometrial adenocarcinomas. Using the same antibody for immunohistochemistry, Shiozawa et al (1997) and Lu et al (1998) report that normal endometrial and cervical cells stain negatively for p16 protein, except for during the proliferative phase, and then only cytoplasmically. Shiozawa et al found 14/41 endometrioid types endometrial tumours were positive for p16, and Lu et al found 23/40 cervical carcinomas were positive for p16. We cannot explain the discrepancy between our positive staining of normal uterine tissues and the negative staining of these two groups.

Recent studies show that aberrant hypermethylation of the CpG islands associated with the *p16* and the *p15* genes might provide an alternative mechanism for gene inactivation, versus the mutation or deletion of these genes. It has been established that the methylation status of the 5′ promoter/enhancer region CpG islands is associated with the level of gene transcription. In normal cells expressing these genes the CpG islands are usually hypomethylated. Acquired hypermethylation has been associated with loss of their mRNA expression (Gonzalez-Zulueta et al, 1995; Herman et al, 1995, 1996; Merlo et al, 1995). Strikingly, aberrant methylation of the *p16* gene was observed frequently not only in neoplasms such as breast and renal cell carcinomas, where homozygous deletion is frequent, but also in the tumours, such as colon and prostate carcinomas, in which homozygous deletions of the *p16* gene are rare (Herman et al, 1995).

The mechanism of aberrant expression of the *p16* gene product in these uterine tumours was evaluated (Table 2). Of nine cervical carcinomas and seven endometrial tumours with aberrant *p16* gene product, four cervical carcinomas and one endometrial carcinoma had homozygous deletion of *p16*, and one endometrial carcinoma had a non-sense mutation in *p16*. Of the remaining five cervical tumours and five endometrial tumours having no deletion or mutation in *p16* gene, two cervical tumours and two endometrial tumours did not express *p16* mRNA. Of these four uterine tumours with loss of *p16* mRNA expression, one squamous cell carcinoma showed hypermethylation within the 5′-CpG island of the *p16* gene. However, the remaining three tumours had no discernable mechanisms to explain their loss of *p16* mRNA expression. Although the specific CpG sites we examined are a frequent target for hypermethylation in human cancers, methylation of other sites might be involved in these remaining three tumours. A mechanism of specific transcription factor repression or deficiency is also possible. It is notable that aberrant expression of p16 protein was observed in five tumours which otherwise showed positive mRNA expression. There may yet exist an alternative translational mechanism, or post-translational degradative mechanism, for the loss of protein expression in these unique tumours.

The association of aberrant p16 expression with clinical stages was evaluated. In cervical carcinomas, the aberrant p16 expression was found in none of one (0%) stage 0 tumours, three of 12 (25%) stage 1 tumours, three of 16 (19%) stage 2 tumours, two of three (67%) stage 3 tumours and one of one (100%) stage 4 tumours. In endometrial carcinomas, the aberrant p16 expression was found in three of 14 (21%) stage 1 tumours, one of eight (13%) stage 2 tumours, two of seven (29%) stage 3 tumours, and none of one (0%) stage 4 tumours. Therefore, there was no association between aberrant p16 expression and clinical stages.

The process of tumorigenesis usually involves the activation of oncogenic and inactivation of tumour suppressor signaling pathways. Activation of the K-*ras* oncogene, frequently mutated in human carcinomas, drives tumour growth forward. Inactivation of some part of the *p53* and *Rb* tumour suppressor pathways is usually required to permit the uncontrolled growth. It is well established that human papillomavirus (HPV) infection is strongly associated with cervical carcinogenesis. The E6 and E7 proteins of the oncogenic strains of HPV bind and inactivate both the p53 and RB proteins respectively. HPV infection can thus often substitute for mutations in both these pathways. For example, Parker et al (1997) found that HPV and p53 overexpression were mutually exclusive in cervical carcinomas, whereas Munirajan et al (1998) found that, of 43 tumours studied with p53 mutations, three of four tumours had HPV infections.

All 33 cervical carcinomas in which we examined the p16 expression by immunohistochemistry had been analysed previously for the presence of HPV infection (Fujita et al, 1992). Therefore, the association of p16 immunoreactivity with HPV infection was evaluated. The aberrant p16 expression was found in

© Cancer Research Campaign 1999

five of 25 (20%) HPV-positive tumours and four of eight (50%) HPV-negative tumours ($P$ = 0.117 by Fisher's exact test). The finding that *p16* inactivation was more frequent in HPV-negative tumours than in HPV-positive tumours (50% vs 20%) follows the predictions based on the molecular biology discussed above. Kim et al (1998) found none of 57 cervical carcinomas had *p16* or *p15* point mutations or homozygous loss of the 9p21 locus.

On the other hand, we previously showed that activation of the K-*ras* oncogene and inactivation of the *p53* tumour suppressor gene occurs in about 31% and 25% of endometrial carcinoma respectively (Enomoto et al, 1993). We examined the p16 expression by immunohistochemistry in 27 of 30 endometrial carcinomas we had analysed previously for the presence of K-*ras* activation and *p53* inactivation (Enomoto et al, 1993). The association of p16 immunoreactivity with K-*ras* activation was evaluated. The aberrant p16 expression was found in two of seven (29%) tumours with K-*ras* activation and in four of 20 (20%) tumours with wild-type K-*ras* sequence ($P$ = 0.50 by Fisher's exact test). The association of p16 immunoreactivity with *p53* inactivation was evaluated. The aberrant p16 expression was found in two of five (40%) tumours with *p53* inactivation and in four of 22 (18%) tumours with wild-type *p53* sequence ($P$ = 0.303). These observations suggest that *p16* inactivation occurs independently to the K-*ras* activation or *p53* inactivation.

The *p15* and *p16* genes possess extensive sequence similarity and both are co-localized on chromosome 9p21. The p15 protein also binds to and inhibits CDK function in vitro, and ectopic expression of p15 inhibits cell growth in vitro (Stone et al, 1995). In spite of the sequence similarity between the *p15* and *p16* genes and their proteins, however, the two proteins have significantly different functions in vivo. The *p15* gene is induced by transforming growth factor (TGF-β), but the *p16* gene is not (Hannon and Beach, 1994). The expression of *p15* is independent of RB regulation, unlike the *p16* gene. Not only the physiological functions, but also their role as tumour suppressor genes, seem to differ between the two genes. Point mutations in *p16* are found in tumours with varying frequency, depending on the tumour type, but are extremely rare in the *p15* gene (Stone et al, 1995). Inactivation of the *p16* gene by hypermethylation occurs in many human solid tumours. However, inactivation of *p15* by CpG-island hypermethylation occurs selectively in leukaemias and gliomas, but not in colon, breast or lung carcinomas (Herman et al, 1995). These previous reports suggest that the *p15* gene is not an important target for inactivation in most human cancers. Our observations, that a *p15* mutation did not occur in any of the 78 uterine tumours, and that homozygous deletion of the *p15* gene was observed in only four cases, all of which also had homozygous deletion of *p16*, suggest that the *p15* gene is not the primary target for deletion in this locus, and *p15* may not play an important role in uterine tumorigenesis.

In conclusion, the *p16* gene is a tumour suppressor gene which is inactivated in a significant number of uterine tumours, although its exact role in uterine tumorigenesis remains to be established. Although the homologous co-deletion of the *p15* gene may occur in a small fraction of uterine cervical and endometrial tumours (4/78), this gene appears not to play an important role in uterine carcinogenesis.

### ACKNOWLEDGEMENTS

This project was funded in part with federal funds from the National Cancer Institute under contract No. NO1-CO-56000. The content of this publication does not necessarily reflect the views or policies of the Department of Health and Human Services, nor does mention of trade names, commercial products, or organizations imply endorsement by the US Government. The publisher or recipient acknowledges the right of the US Government to retain a non-exclusive, royalty-free license in and to any copyright covering this article.

### REFERENCES

Enomoto T, Weghorst CM, Inoue M, Tanizawa O and Rice JM (1991) K-*ras* activation occurs frequently in mucinous adenocarcinomas and rarely in other common epithelial tumors of the human ovary. *Am J Pathol* **139**: 777–785

Enomoto T, Fujita M, Inoue M, Rice JM, Nakajima R, Tanizawa O and Nomura T (1993) Alterations of the *p53* tumor suppressor gene and its association with activation of the c-K-*ras*-2 protooncogene in premalignant and malignant lesions of the human uterine endometrium. *Cancer Res* **53**: 1883–1888

Foulkes WD, Flanders TY, Pollock PM and Hayward NK (1997) The *CDKN2A* (*p16*) gene and human cancer. *Mol Med* **3**: 5–20

Fujita M, Inoue M, Tanizawa O, Iwamoto S and Enomoto T (1992) Alterations of the *p53* gene in human primary cervical carcinoma with and without human papillomavirus infection. *Cancer Res* **52**: 5323–5328

Fuqua SAW, Falette NF and McGuire WL (1990) Sensitive detection of estrogen receptor RNA by polymerase chain reaction assay. *J Natl Cancer Inst* **82**: 858–861

Gonzalez-Zulueta M, Bender CM, Yang AS, Nguyen TW, Beart R, Van Tornout JM and Jones PA (1995) Methylation of the 5′ CpG island of the *p16*/*CDKN2* tumor suppressor gene in normal and transformed human tissues correlates with gene silencing. *Cancer Res* **55**: 4531–4535

Guan K-L, Jenkins CW, Li Y, Nichols MA, Wu X, O'Keefe CL, Matera AG and Xiong Y (1994) Growth suppression by *p18*, a p16$^{INK4/MTS1}$-and p14$^{INK4B/MTS2}$-related CDK6 inhibitor, correlates with wild-type pRb function. *Genes Dev* **8**: 2939–2952

Hannon GJ and Beach D (1994) p15$^{INK4B}$ is a potential effector of TGF-β-induced cell cycle arrest. *Nature (Lond)* **371**: 257–261

Hatta Y, Hirama T, Takeuchi S, Lee E, Pham E, Miller CW, Strohmeyer T, Wilczynski SP, Melmed S and Koeffler HP (1995) Alterations of the p16 (MTS1) gene in testicular, ovarian, and endometrial malignancies. *J Urol* **154**: 1954–1957

Hengstschläger M, Hengstschläger-Ottnad E, Pusch O and Wawra E (1996) The role of p16 in the E2F-dependent thymidine kinase regulation. *Oncogene* **12**: 1635–1643

Herman JG, Jen J, Merlo A and Baylin SB (1996) Hypermethylation-associated inactivation indicates a tumor suppressor role for *p15*$^{INK4B}$. *Cancer Res* **56**: 722–727

Herman JG, Merlo A, Mao L, Lapidus RG, Issa J-PJ, Davidson NE, Sidransky D and Baylin SB (1995) Inactivation of the *CDKN2*/*p16*/*MTS1* gene is frequently associated with aberrant DNA methylation in all common human cancers. *Cancer Res* **55**: 4525–4530

Hirama T, Miller CW, Wilczynski SP and Koeffler HP (1996) *p16* (CDKN2/cyclin-dependent kinase-4 inhibitor/multiple tumor suppressor-1) gene is not altered in uterine cervical carcinomas or cell lines. *Modern Pathol* **9**: 26–31

Jen J, Harper JW, Bigner SH, Bigner DD, Papadopoulos N, Markowitz S, Willson JKV, Kinzler KW and Vogelstein B (1994) Detection of *p16* and *p15* genes in brain tumors. *Cancer Res* **54**: 6353–6358

Kamb A, Gruis NA, Weaver-Feldhaus J, Liu Q, Harshman K, Tavtigian SV, Stockert E, Day RS III, Johnson BE and Skolnick MH, (1994) A cell cycle regulator potentially involved in genesis of many tumor types. *Science* **264**: 436–440

Kelly MJ, Otterson GA, Kaye FJ, Popescu NC, Johnson BE and Dipaulo JA (1995) *CDKN2* in HPV-positive and HPV-negative cervical-carcinoma cell lines. *Int J Cancer* **63**: 226–230

Kim JW, Namkoong SE, Ryu SW, Kim HS, Shin JW, Lee JM, Kim DH and Kim IK (1998) Absence of p15(INK4B) and p16(INK4A) gene alterations in primary cervical carcinoma tissues and cell lines with human papillomavirus infection. *Gynecol Oncol* **70**: 75–79

Lo K-W, Cheung S-T, Leung S-F, Van Hasselt A, Tsang Y-S, Mak K-F, Chung Y-F, Woo JKS, Lee JCK and Huang DP (1996) Hypermethylation of the *p16* gene in nasopharyngeal carcinoma. *Cancer Res* **56**: 2721–2725

Lu X, Toki T, Konishi I, Nikaido T and Fujii S (1998) Expression of p21WAF1/CIP1 in adenocarcinoma of the uterine cervix: a possible immunohistochemical marker of a favorable prognosis. *Cancer* **82**: 2409–2417

© Cancer Research Campaign 1999

Lukas J, Otzen Petersen B, Holm K, Bartek J and Helin K (1996) Deregulated expression of E2F family members induces S-phase entry and overcomes $p16^{INK4A}$-mediated growth suppression. *Mol Cell Biol* **16**: 1047–1057

Marchetti A, Buttitta F, Pellegrini S, Bertacca G, Chella A, Carnicelli V, Tognoni V, Filardo A, Angeletti A and Bevilacqua G (1997). Alterations of *p16 (MTS1)* in node-positive non-small cell lung carcinomas. *J Pathol* **181**: 178–182

Merlo A, Herman JG, Mao L, Lee DJ, Gabrielson E, Burger PC, Baylin SB and Sidransky D (1995) 5′ CpG island methylation is associated with transcriptional silencing of the tumour suppressor *p16*/*CDKN2*/*MTS1* in human cancers. *Nat Med* **1**: 686–692

Munirajan AK, Kannan K, Bhuvarahamurthy V, Ishida I, Fujinaga K, Tsuchida N and Shanmugam G (1998) The status of human papillomavirus and tumor suppressor genes p53 and p16 in carcinomas of uterine cervix from India. *Gynecol Oncol* **69**: 205–209

Nobori T, Miura K, Wu DJ, Lois A, Takabayashi K and Carson DA (1994) Deletions of the cyclin-dependent kinase-4 inhibitor gene in multiple human cancers. *Nature (Lond)* **368**: 753–756

Owen D and Küehn LC (1987) Noncoding 3′ sequences of the transferrin receptor gene are required for mRNA regulation by iron. *EMBO J* **6**: 1287–1293

Parker MF, Arroyo GF, Geradts J, Sabichi AL, Park RC, Taylor RR and Birrer MJ (1997) Molecular characterization of adenocarcinoma of the cervix. *Gynecol Oncol* **64**: 242–251

Peiffer SL, Bartsch D, Whelan AJ, Mutch DG, Herzog TJ and Goodfellow PJ (1995) Low frequency of *CDKN2* mutation in endometrial carcinomas. *Mol Carcinog* **13**: 210–212

Pollock PM, Pearson JV and Hayward NK (1996) Compilation of somatic mutations of the *CDKN2* gene in human cancers, non-random distribution of base substitutions. *Gene Chromosomes Cancer* **15**: 77–88

Serrano M, Haanon GJ and Beach D (1993) A new regulatory motif in cell-cycle control causing specific inhibition of cyclin D/CDK4. *Nature (Lond)* **366**: 704–707

Sheffield VC, Beck JS, Kwitek AE, Sandstrom DW and Stone EM (1993) The sensitivity of single-strand conformation polymorphism analysis for the detection of single base substitutions. *Genomics* **16**: 325–332

Shiozawa T, Nikaido T, Shimizu M, Zhai Y and Fujii S (1997) Immunohistochemical analysis of the expression of cdk4 and $p16^{INK4}$ in human endometrioid-type endometrial carcinoma. *Cancer* **80**: 2250–2256

Stone S, Jiang P, Dayananth P, Tavtigian SV, Katcher H, Parry D, Peters G and Kamb A (1995) Complex structure and regulation of the *p16* (*MTS1*) locus. *Cancer Res* **55**: 2988–2994

Washimi O, Nagatake M, Osada H, Ueda R, Koshikawa T, Seki T, Takahashi T and Takahashi T (1995) In vivo occurrence of p16 (MTS1) and p15 (MTS2) alterations preferentially in non-small cell lung cancers. *Cancer Res* **55**: 514–517

Wong YF, Chung TKH, Cheung TH, Nobori T, Yim SF, Lai KWH, Yu AL, Diccianni MB, Li TZ and Chang AMZ (1997) $p16^{INK4}$ and $p15^{INK4B}$ alterations in primary gynecologic malignancy. *Gynecol Oncol* **65**: 319–324

*© Cancer Research Campaign 1999*
